# Patient preferences and choices as a reflection of trust—A cluster analysis comparing postsurgical perceptions in a private and a public hospital

**DOI:** 10.1111/hex.13487

**Published:** 2022-07-14

**Authors:** Royi Barnea, Aviad Tur‐Sinai, Osnat Levtzion‐Korach, Yossi Weiss, Orna Tal

**Affiliations:** ^1^ Assuta Health Services Research Institute Assuta Medical Centers Tel‐Aviv Israel; ^2^ School of Health Systems Management Netanya Academic College Netanya Israel; ^3^ Department of Health Systems Management The Max Stern Yezreel Valley College Yezreel Valley Israel; ^4^ School of Nursing University of Rochester Medical Center Rochester New York USA; ^5^ Shamir Medical Center (Assaf Harofeh) Zerifin Israel; ^6^ Ariel University Ariel Israel; ^7^ Israeli Center for Emerging Technologies (ICET) Tel Aviv Israel; ^8^ Department of Management Bar Ilan University Ramat Gan Israel

**Keywords:** cluster analysis, commitment, patient's empowerment, patient's preferences, patient's trust, private hospital, public hospital, surgical procedure

## Abstract

**Background:**

Active participation of patients in managing their medical treatment is a major component of the patient empowerment process and may contribute to better clinical outcomes. Patient perceptions and preferences affect the patient–physician encounter in a variety of dimensions, such as patient autonomy, freedom of choice and trust in the healthcare system. The Israeli healthcare system is mostly publicly funded, with additional private healthcare services for surgery and other medical treatments. The aim of this study was to compare the perceptions and preferences of patients in the public and private hospitals in Israel.

**Methods:**

A cross‐sectional study among 545 individuals who had surgical procedures at two hospitals in Israel (one public and one private). A structured questionnaire comprising 23 items was used to collect perceptions via personal telephone interviews. The responses were categorized into five clusters and compared by type of health services provider (public vs. private) and sociodemographic characteristics (gender, age and education level).

**Results:**

A hierarchical cluster analysis methodology identified five conceptual groups: trust, concern towards medical errors, dialogue between medical staff and the patient/patient's family, confidentiality and staff bias towards more informed patients, or those with supportive families. Four main themes that highlight patients' preferences were found: physical conditions, personal empowerment and perceived autonomy, patient experience and patient–provider encounter communication. Significant differences between the private and the public healthcare systems were found in four clusters: trust and patient care, patient's concerns, the extent of explanation and medical staff's commitment. Differences secondary to sociodemographic parameters were noticed: patients treated at the private hospital scored significantly higher items of trust, medical staff caring and the importance of choosing their treating surgeon, while patients treated at the public hospital scored higher staff commitment to the patient than those treated at the private hospital.

**Conclusions:**

The study revealed the perceptions underlying the decisions of patients to undergo surgical procedures in public or private hospitals. Mutual learning could pave the way to better patient–physician encounters.

**Patient or Public Contribution:**

Patients from the two hospitals were involved in this study by responding to the questionnaire. The data presented is based on the patient's responses.

## 1 INTRODUCTION

One of the evolving trends in modern healthcare systems is the growing involvement and active participation of patients in managing their medical treatment.^1^, ^2^, ^3^ This is a major component of the patient empowerment process that highlights the perception that patient preferences may lead to better clinical outcomes.^4^, ^5^ These preferences may be related to several fundamental issues, including patients' trust in the healthcare system, autonomy and freedom of choice, the extent of knowledge/information provided to patients and shared decision‐making.^6^, ^7^

Patients' trust in their healthcare professionals is fundamental to effective treatment in clinical practice. The achievement of successful medical care depends on patients' trust in their physicians: believe they are competent, take appropriate responsibility and control and are eager to provide their patients' welfare at the highest priority.^8^ Patients were more satisfied with the treatment, showed more beneficial health behaviours, informed less symptoms and reported a higher quality of life when they have higher trust in their healthcare professional.^9^

Choice and patient autonomy are two of the most discussed concepts in bioethics. The value of choice in healthcare has several aspects: choice allows individuals to take control and to make their life their own, it is also valuable for instrumental reasons, because if an individual is sufficiently informed, he/she may be the best judge of their own best interests. In addition, it has been suggested that simply the option of having a choice, makes it valuable^10^, ^11^ thus elements of autonomy, control and personalized care play a role in the decision‐making process.

Shared decision‐making is defined as a ‘process jointly shared by patients and their healthcare provider. It aims at helping patients play an active role in decisions concerning their health, which is the ultimate goal of patient‐centred care’.^12^ This concept has been associated with patient autonomy^13^ and empowerment.^14^ However, to practice shared decision‐making, the healthcare provider must first provide relevant and clear information to the patients so that they would be able to consider their options and participate in the shared decision‐making process,^15^ embedding trust, freedom of choice and cooperation, beyond individual concerns. All of these issues reflect the importance of the patient‐centred care approach in modern healthcare. The Israeli public healthcare system is mostly funded by the government, delivering care through four health maintenance organizations to any citizen needing medical attention regardless of the ability to pay.^16^ However, a continuing lack of resources in the public healthcare system has led to prolonged waiting times for elective surgeries. As a result, patients turn to private healthcare services for surgery and other medical treatments.^17^, ^18^ These private services are paid for by the patient (out‐of‐pocket) or through private health insurance.^19^ Patients who plan to have surgery in the public healthcare system cannot choose their surgeon, while patients who have surgery through private healthcare services have greater freedom of choice as they can choose both their surgeon and hospital.^20^ This phenomenon raises serious issues relating to equity, the relation between the ability to pay and health services consumption and patient autonomy.

In addition to their different budgetary sources, the Israeli public and private healthcare systems differ in seniority of caregivers, targeted teaching time and in the performance volume of selected types of procedures, resulting in an overload of the public health system. It is perceived that in private hospitals, the presurgical process and particularly the patient–surgeon encounter is more detailed in terms of the information provided to patients and their families, greater attention given to concerns raised by patients during and following their hospital stay, and the extent of commitment of medical staff.

The patient‐medical team encounter is a crucial junction in determining patient trust, treatment compliance and enhanced quality of care.^21^, ^22^ In a study performed in Sweden, factors such as dialogue, information, attention and participation affected patients' sense of involvement and control during an emergency room visit, which, in turn, were linked to their experiences of care and to being seen as individuals.^22^ Anhang Price et al.^23^ indicated that better patient care experiences are associated with higher levels of adherence to recommended prevention and treatment processes, better clinical outcomes, better patient safety within hospitals and less healthcare utilization.

To understand patients' preferences to undergo surgical procedures in public or private hospitals, this study compared the perceptions and experiences of patients who had surgery in one public and one private hospital in Israel.

## 2 MATERIALS AND METHODS

### 2.1 Study design and setting

This cross‐sectional study was performed in April–September 2020 among individuals who had surgical procedures in one of two hospitals in Israel: a public (government‐owned) hospital and a privately owned hospital. Both hospitals provide a broad range of surgical procedures. The two hospitals mainly provide health services to individuals residing in central Israel; therefore, their patients' demographic characteristics are similar. Additionally, both hospitals have a high volume of surgical activity with no patient selection. The selected procedures for this study were identical for both hospitals and the main difference between them is the type of ownership (public vs. private).

### 2.2 Sampling technique

The 10 most frequent elective surgical procedures performed in both hospitals among all patients admitted to surgical procedures (both children and adults) in the year preceding the study were identified Then, all persons who had undergone at least one of these surgical procedures in January–March 2020 were identified: 2415 in the public hospital and 2118 in the private hospital. To attain the fullest possible understanding of the scientific issue, a target was set of at least 10% (250 people) of those who had surgery in each hospital.

### 2.3 Ethical issues

The study's ethics, procedure, and research tool (questionnaire) were approved by the ethics committees of each participating hospital (reference numbers 0108‐19‐ASF for the public hospital and 0034‐19‐ASMC for the private one). All participants received information on the study's purpose, the confidentiality of the information and the right to revoke their participation without prior justification, and provided their consent for participation.

### 2.4 Questionnaire and data collection

A structured questionnaire was used to collect perceptions via personal telephone interviews. The questionnaire was based on grounded theory and comprised 23 items. The questionnaire was developed based on a previous study that was conducted by the authors,^8^ deliberated by a steering committee, and a pilot test was conducted to validate the questionnaire.

The participants were asked to rate their agreement with each item on a scale ranging from 1 (the smallest extent) to 10 (the largest extent). The items addressed a series of issues relating to trust between the participant and the hospital's medical staff, the medical staff's commitment to the patient, the extent of explanation given about the procedure, the cooperation between the patient, the patient's family and the medical staff, concern about mistakes and inaccuracies in medical decisions and questions about the patient's ability to control the course of his/her inpatient stay and if he/she was aware of the various stages of medical care provided (Tables 2, 3, 4). The reliability of the questionnaire was tested, showing a Cronbach's *α* of .864. The participants were also asked a set of questions on their sociodemographic (gender, age, marital status, country of birth, religion), health (self‐rated health status, extent of physical limitations that impair daily functioning, medical insurance coverage) and economic background (level of education, current employment status, household income). Each participant answered the phone questionnaire at his/her convenience; unlimited response time was allowed for each question.

### 2.5 Statistical analysis

For each of the questionnaire items, means and standard deviations were calculated in accordance with the type of health services provider (public or private hospital) and compared. The answers were also compared among subgroups parsed by sociodemographic characteristics: gender, age group (<40, 40–59, ≥60 years) and education level (≤12 and >12 years). An independent *t*‐test was used to determine the statistical variance between reference groups. Statistical significance was deemed if the *p* value was less than .05.

### 2.6 Cluster analysis

To identify conceptual groups of the questionnaire items, a hierarchical cluster analysis methodology was used.^16^, ^17^ This analysis was carried out with the aim of detecting, within the 23 trust questions for which data were available, the presence of groups of cases that are both similar (i.e., presenting ‘maximum similarity’) within each group and, at the same time, as different as possible from the other groups (i.e., reflecting the ‘highest diversity’ between clusters). For this purpose, the complete linkage (or ‘furthest neighbour’) method was used and the clusters were created by adding, in each step, the nearest case to all others already present in the specific group. The squared Euclidean distance between cases was used to give progressively greater weight to cases that were beyond a defined distance. Two indicators were used for this analysis: one for the public health system and one for the private health system, represented by the average value obtained from the answers of persons who had a surgical procedure in each health system.

## 3 RESULTS

A total of 545 participants were included in the study: 287 (52.7%) had surgery in the public hospital (mean age: 51.8 years) and 258 (47.3%) had surgery in the private hospital (mean age: 55.2 years). In both groups, most participants were ≥60 years old, and a relatively few were under 40 years of age. About 55% of participants in both hospital types were women. In contrast, two‐thirds of the participants who had surgery in the public hospital had basic education only (up to 12 years of education) whereas two‐thirds of those who had surgery in the private hospital had more than 12 years of education. The study population's sociodemographic characteristics are summarized in Table 1.

**Table 1:** Participant demographics

Variable	Type of service provider			All (*N* = 545)
	Private hospital (*N* = 258)	Public hospital (*N* = 287)	*p* Value	
Age (years), mean (SD)	55.23 (16.38)	51.82 (18.42)	**.0325**	53.43 (17.55)
Age group, *n* (%)				
<40	46 (17.83)	72 (25.09)		118 (21.65)
40–59	71 (27.52)	79 (27.53)	.095	150 (27.52)
>60	141 (54.65)	136 (47.39)		277 (50.83)
Gender, *n* (%)				
Male	115 (44.57)	123 (42.86)	.724	238 (43.67)
Female	143 (55.43)	164 (57.14)		307 (56.33)
Education level, *n* (%)				
≤12 years	84 (32.56)	189 (65.85)	**.000**	273 (50.09)
>12 years	174 (67.44)	98 (34.15)		272 (49.91)

*Note*: Bold formatting of *p* values indicates significance (*p* < .05).

Abbreviation: SD, standard deviation.

## 4 AN OVERVIEW OF THE FINDINGS

Differences were observed in the perceptions and views of postoperative patients who had surgery at a public hospital compared to those who had surgery at a private hospital. For example, responders perception was that, in the private hospital, medical staff showed higher accountability (more among women and young patients), had better credentials (by both genders), assured the correct prescription to avoid error (more among young patients) and displayed a higher trust, thus tending to highly recommend the private pathway to their friends and relatives (more among women) in spite of economic issues (more among men, elderly and less educated patients). The importance of choosing their surgeon was emphasized by both genders.

A thorough analysis revealed sociodemographic parameters are a key factor in shaping patients' preferences. In our study, certain sociodemographic parameters differ among the subpopulations: patients of the private hospital were older and more educated and may also correlate with the economic resources available for medical treatment, thus influencing their choices.

Interestingly, women who were treated at the private hospital scored higher to the extent of explanation and cooperation among patients, their families and the medical staff. This observation was not found in men who had surgery at a private hospital.

Stratification of the responders by age showed that young patients also scored higher in the extent of explanation and cooperation between the patient/patient's family and the medical staff.

We also examined the relationship between patient education and patient experience. Across all levels of education, patients who had surgery at the private hospital gave higher scores to the extent of explanation and cooperation between the patient/patient's family and the medical staff compared to those who had surgery at the public hospital (Table 4). Public healthcare patients reported higher levels of medical staff commitment compared to those treated at the private hospital, regardless of their education levels. In almost all other dimensions, such as patient concerns and additional considerations during their hospital stay and the extent of explanation and cooperation between the patient/patient's family and the medical staff—no significant effect was found between the private and the public healthcare systems, implying similar patient experience, journey and possibly, similar trust levels while examining these components.

### 4.1 Perceived degree of patient trust and patient care characteristics

*The first cluster* focuses on the degree of patient trust and patient care characteristics; it comprised seven questions that reflect trust in the entire process that the patient went through at the hospital such as; free choice; staff attention; clear understandable explanations; recommendation of the hospital to friends and relatives; true staff concern for patient's health; and staff good medical credentials.

Overall, the participants perceived that the medical staff looked after their health to a great extent, with those treated at the private hospital giving significantly higher mean scores than those treated at the public hospital (9.36 vs. 8.99, *p* = .0149). Similarly, women treated at the private hospital gave significantly higher mean scores than those treated at the public hospital (Table 2). This difference was not observed for men. Young participants (<40) who had surgery at the private hospital, as well as participants with academic education, also perceived that the medical staff looked out for their health more than their counterparts treated at the public hospital (9.61 vs. 8.56, *p* = .0056 and 9.5 vs. 8.81. *p* = .0444, respectively), while this difference was not observed for older participants and for participants with ≤12 years of education (Table 3).

In general, the participants perceived that the medical credentials of the medical staff were appropriate for any situation, but those treated at the private hospital gave significantly higher mean scores than those treated at the public hospital (9.29 vs. 8.80, *p* = .0013). This difference was also observed among women but not among men (Table 2). Similarly, women and participants aged <40 years who had surgery at the private hospital gave significantly higher mean scores to this statement than those treated at the public hospital. This difference was not observed for men, participants 40 and above (Tables 2, 3). Education level did not affect this observation (Table 4).

**Table 2:** Survey core variables by type of service provider and gender

Question		All (*N* = 545) Mean (SD)			Females (*N* = 307) Mean (SD)			Males (*N* = 238) Mean (SD)		
		Private hospital (*N* = 258)	Public hospital (*N* = 287)	*p* Value	Private hospital (*N* = 143)	Public hospital (*N* = 164)	*p* Value	Private hospital (*N* = 115)	Public hospital (*N* = 123)	*p* Value
*Cluster 1: The degree of trust and characteristics of patient care given by the medical staff*										
1.	To what extent did the medical staff look out for your health?	9.36 (1.38)	8.99 (1.97)	**.0149**	9.38 (1.38)	8.75 (2.33)	**.0057**	9.31 (1.41)	9.31 (1.32)	.9756
5.	To what extent did you feel that the medical credentials of the medical staff were good for any situation?	9.29 (1.39)	8.80 (1.99)	**.0013**	9.29 (1.47)	8.71 (2.30)	**.0119**	9.31 (1.31)	8.97 (1.35)	.0646
10.	To what extent were you trustful in placing your life in the hands of the hospital's medical staff?	9.27 (1.32)	8.81 (1.65)	**.0007**	9.27 (1.39)	8.63 (1.87)	**.0014**	9.24 (1.26)	9.02 (1.31)	.2081
11.	To what extent is it important for you to choose your treating surgeon and to know that only she/he is caring for you?	9.70 (1.23)	7.75 (3.23)	**<.0001**	9.83 (0.85)	7.85 (3.28)	**<.0001**	9.51 (1.61)	7.64 (3.18)	**<.0001**
12.	To what extent did the medical staff listen to you and address your questions and concerns?	9.22 (1.60)	9.00 (2.00)	.1741	9.24 (1.59)	8.87 (2.28)	.1232	9.18 (1.65)	9.16 (1.56)	.9093
13.	To what extent did you find the explanations that you received from the medical staff during your hospital stay clear and understandable?	9.33 (1.30)	9.04 (2.05)	.0608	9.47 (1.10)	8.86 (2.40)	**.0078**	9.12 (1.51)	9.30 (1.36)	.3595
17.	Should it become necessary, to what extent would you recommend this hospital to friends and relatives?	9.35 (1.66)	8.96 (2.21)	**.0261**	9.45 (1.69)	8.78 (2.54)	**.0105**	9.20 (1.63)	9.19 (1.65)	.9476
*Cluster 2: The patients' concerns and additional considerations during their hospital stay*										
2.	To what extent did you consider your financial situation when you were hospitalized?	2.65 (2.94)	2.33 (2.71)	.2066	2.67 (2.99)	2.53 (2.91)	.6960	2.73 (2.96)	2.07 (2.42)	.0769
3.	To what extent were you concerned that the staff might make a mistake in its medical decisions?	2.89 (2.99)	2.96 (2.87)	.7982	2.91 (3.09)	2.92 (2.92)	.9776	2.82 (2.83)	2.97 (2.75)	.7704
4.	If the staff asks you to take part in a study, to what extent would you fear that they would care more about the study and less about what matters to you?	3.98 (3.51)	3.42 (2.50)	**.0422**	4.07 (3.51)	3.61 (2.69)	.2236	3.70 (3.42)	3.11 (2.09)	.1357
*Cluster 3: The extent of explanation and cooperation between the patient/patient's family and the medical staff*										
6.	To what extent did you feel that the medical staff allowed you to say things that were important to you?	8.95 (1.98)	8.94 (2.05)	.9632	8.89 (2.06)	8.83 (2.25)	.8092	9.04 (1.91)	9.18 (1.56)	.5464
7.	To what extent does the hospital staff make sure that the prescription for medication that you've been given is correct and accurate?	8.59 (2.90)	9.02 (2.10)	.0606	8.87 (2.64)	9.10 (2.14)	.4181	8.28 (3.15)	8.96 (1.93)	.0562
8.	To what extent did the medical staff at the hospital present you with options of care so that you could choose freely?	8.17 (3.13)	8.42 (2.29)	.3056	7.88 (3.42)	8.37 (2.53)	.1702	8.52 (2.77)	8.47 (1.97)	.8775
15.	To what extent did you feel that you knew what the next stage of care would be?	8.85 (2.12)	8.84 (2.27)	.9828	8.90 (2.16)	8.69 (2.52)	.4632	8.73 (2.10)	9.02 (1.90)	.2910
16.	To what extent did you feel that you were included in decisions and that your preferences were considered?	8.38 (2.62)	8.10 (2.46)	.2074	8.52 (2.64)	8.05 (2.63)	.1324	8.16 (2.62)	8.12 (2.23)	.9005
21.	To what extent do you agree with this sentence: ‘The doctor–patient relationship is like a contract/a partnership meant to attain health’?	8.89 (2.23)	8.93 (2.42)	.8390	8.94 (2.27)	9.01 (2.29)	.8000	8.80 (2.22)	9.03 (2.37)	.4792
22.	To what extent do you believe that the patient's family shares responsibility for care?	7.95 (3.15)	8.61 (2.61)	**.0118**	8.24 (3.03)	8.56 (2.61)	.3358	7.62 (3.24)	8.88 (2.31)	**.0013**
23.	To what extent do you agree with this sentence: ‘Four players share responsibility for healthcare: the doctor, the patient, the family, and the hospital’?	8.94 (2.27)	8.78 (2.65)	.4669	9.21 (1.99)	8.89 (2.52)	.2465	8.60 (2.55)	8.83 (2.58)	.5206
*Cluster 4: The way the staff handles medical information*										
9.	To what extent is it important for you that the staff keep your information confidential?	9.07 (2.29)	9.34 (1.70)	.1247	9.29 (2.09)	9.43 (1.57)	.5160	8.85 (2.43)	9.21 (1.89)	.2236
14.	To what extent did you feel that the medical staff coordinated and cooperated with one another in its work (e.g., in sharing information with one another)?	9.15 (1.91)	9.06 (1.88)	.6215	9.17 (1.95)	8.82 (2.26)	.1702	9.11 (1.89)	9.39 (1.61)	.2055
*Cluster 5: The commitment strength of medical staff to the patient*										
18.	‘To what extent does the medical staff at the hospital tend to be committed to the patient who makes an effort to be involved in care?’	6.35 (3.94)	8.46 (2.50)	**<.0001**	6.79 (3.88)	8.36 (2.65)	**.0001**	5.81 (3.98)	8.65 (2.20)	**<.0001**
19.	To what extent does the medical staff at the hospital tend to be more committed to a patient who has a family?	4.97 (4.03)	8.05 (2.92)	**<.0001**	5.48 (4.16)	8.09 (2.94)	**<.0001**	4.16 (3.71)	8.11 (2.78)	**<.0001**
20.	‘To what extent does the medical staff at the hospital tend to be more committed to a well‐educated patient?’	3.73 (3.86)	6.91 (3.35)	**<.0001**	3.54 (3.92)	7.06 (3.44)	**<.0001**	3.88 (3.74)	6.86 (3.14)	**<.0001**

*Note*: Bold formatting of *p* values indicates significance (*p* < .05).

Abbreviation: SD, standard deviation.

**Table 3:** Survey core variables by type of service provider and age category

Question		Young (<40 years) (*N* = 118) Mean (SD)			Middle‐aged (40–59 years) (*N* = 150) Mean (SD)			Elderly (>60 years) (*N* = 277) Mean (SD)		
		Private hospital (*N* = 46)	Public hospital (*N* = 72)	*p* Value	Private hospital (*N* = 71)	Public hospital (*N* = 79)	*p* Value	Private hospital (*N* = 141)	Public hospital (*N* = 136)	*p* Value
*Cluster 1: The degree of trust and characteristics of patient care given by the medical staff*										
1.	To what extent did the medical staff look out for your health?	9.61 (0.98)	8.56 (2.40)	**.0056**	9.25 (1.65)	8.89 (2.06)	.2481	9.34 (1.33)	9.33 (1.54)	.9642
5.	To what extent did you feel that the medical credentials of the medical staff were good for any situation?	9.35 (1.12)	8.50 (2.12)	**.0138**	9.20 (1.74)	8.63 (2.21)	.0879	9.33 (1.28)	9.09 (1.73)	.2216
10.	To what extent were you trustful in placing your life in the hands of the hospital's medical staff?	8.91 (1.81)	8.43 (1.61)	.1467	9.01 (1.59)	8.64 (2.00)	.2115	9.53 (0.82)	9.15 (1.36)	**.0075**
11.	To what extent is it important for you to choose your physician–caregiver and to know that only she or he is caring for you?	9.52 (1.55)	6.84 (3.68)	**<.0001**	9.73 (1.13)	7.89 (3.00)	**<.0001**	9.74 (1.17)	8.19 (3.00)	**<.0001**
12.	To what extent did the medical staff listen to you and address your questions and concerns?	9.47 (1.01)	8.57 (2.37)	**.0182**	9.17 (1.84)	8.91 (1.85)	.3901	9.15 (1.64)	9.31 (1.81)	.4831
13.	To what extent did you find the explanations that you received from the medical staff during your hospital stay clear and understandable?	9.39 (0.95)	8.82 (2.24)	.1125	9.30 (1.60)	8.83 (2.04)	.1229	9.32 (1.24)	9.31 (1.91)	.9404
17.	Should it become necessary, to what extent would you recommend this hospital to friends and relatives?	9.52 (1.19)	8.57 (2.69)	**.0285**	9.44 (1.48)	8.77 (2.26)	**.0382**	9.24 (1.88)	9.32 (1.77)	.7396
*Cluster 2: The patients' concerns and additional considerations during their hospital stay*										
2.	To what extent did you consider your financial situation when you were hospitalized?	2.09 (2.47)	2.24 (2.59)	.7698	2.69 (3.02)	2.60 (3.05)	.8579	2.81 (3.04)	2.22 (2.56)	.0998
3.	To what extent were you concerned that the staff might make a mistake in its medical decisions?	2.96 (2.83)	3.21 (2.99)	.6469	2.93 (2.98)	3.63 (3.11)	.1655	2.85 (3.06)	2.38 (2.51)	.1825
4.	If the staff asks you to take part in a study, to what extent would you fear that they would care more about the study and less about what matters to you?	3.38 (3.18)	3.62 (2.41)	.6573	4.03 (3.30)	3.95 (2.63)	.8677	4.14 (3.70)	2.95 (2.39)	**.0041**
*Cluster 3: The extent of explanation and cooperation between the patient/patient's family and the medical staff*										
6.	To what extent did you feel that the medical staff allowed you to say things that were important to you?	9.24 (1.38)	8.61 (2.22)	.0894	8.83 (2.13)	8.96 (1.86)	.6966	8.92 (2.07)	9.14 (2.04)	.4013
7.	To what extent does the hospital staff make sure that the prescription for medication that you've been given is correct and accurate?	9.53 (1.55)	8.58 (2.58)	**.0377**	7.36 (3.77)	9.23 (1.52)	**.0001**	8.96 (2.47)	9.15 (2.07)	.5282
8.	To what extent did the medical staff at the hospital present you with options of care so that you could choose freely?	9.02 (2.27)	8.07 (2.54)	**.0428**	7.99 (3.41)	8.44 (2.15)	.3358	7.98 (3.20)	8.62 (2.22)	.0723
15.	To what extent did you feel that you knew what the next stage of care would be?	8.86 (2.13)	8.37 (2.64)	.3070	8.90 (2.05)	8.82 (2.24)	.8134	8.81 (2.17)	9.14 (2.01)	.2232
16.	To what extent did you feel that you were co‐opted into decisions and that your preferences were taken into account?	8.86 (1.95)	7.43 (2.80)	**.0044**	8.44 (2.66)	7.99 (2.38)	.2774	8.19 (2.79)	8.57 (2.20)	.2426
21.	To what extent do you agree with this sentence: ‘The doctor–patient relationship is like a contract/a partnership meant to attain health’?	8.67 (2.23)	8.95 (2.25)	.5183	9.11 (1.83)	9.10 (1.85)	.9524	8.83 (2.44)	8.81 (2.81)	.9531
22.	To what extent do you believe that the patient's family shares responsibility for care?	7.82 (3.09)	8.97 (2.04)	**.0183**	7.82 (3.09)	8.77 (2.27)	**.0339**	8.08 (3.24)	8.28 (3.07)	.6292
23.	To what extent do you agree with this sentence: ‘Four players share responsibility for healthcare: the doctor, the patient, the family, and the hospital’?	8.61 (2.55)	9.06 (2.27)	.3241	8.82 (2.20)	8.66 (2.72)	.6986	9.15 (2.19)	8.68 (2.83)	.1646
*Cluster 4: The way the staff handles medical information*										
9.	To what extent is it important for you that the staff keep your information confidential?	9.35 (1.72)	9.16 (1.98)	.5819	9.17 (2.22)	9.49 (1.34)	.2835	8.92 (2.49)	9.36 (1.74)	.1116
14.	To what extent did you feel that the medical staff coordinated and cooperated with one another in its work (e.g., in sharing information with one another)?	9.42 (1.32)	8.85 (2.02)	.0988	9.14 (2.00)	8.93 (1.92)	.5241	9.05 (2.03)	9.27 (1.76)	.3732
*Cluster 5: The commitment strength of medical staff to the patient*										
18.	‘To what extent does the medical staff at the hospital tend to be committed to the patient who makes an effort to be involved in care?’	6.55 (4.05)	8.41 (2.21)	**.0023**	6.03 (3.99)	8.70 (2.34)	**<.0001**	6.47 (3.91)	8.32 (2.77)	**.0001**
19.	To what extent does the medical staff at the hospital tend to be more committed to a patient who has a family?	5.38 (4.07)	8.27 (2.34)	**<.0001**	4.57 (4.06)	7.80 (3.11)	**<.0001**	5.06 (4.01)	8.08 (3.10)	**<.0001**
20.	‘To what extent does the medical staff at the hospital tend to be more committed to a well‐educated patient?’	4.15 (4.09)	7.16 (3.09)	**<.0001**	3.87 (3.92)	6.99 (3.27)	**<.0001**	3.51 (3.75)	6.69 (3.56)	**<.0001**

*Note*: Bold formatting of *p* values indicates significance (*p* < .05).

Abbreviation: SD, standard deviation.

**Table 4:** Survey core variables by type of service provider and education level

Question		Basic education (≤12 years) (*N* = 273) Mean (SD)			Academic education (>12 years) (*N* = 272) Mean (SD)		
		Private hospital (N = 84)	Public hospital (*N* = 189)	*p* Value	Private hospital (*N* = 174)	Public hospital (*N* = 98)	*p* Value
*Cluster 1: The degree of trust and characteristics of patient care given by the medical staff*							
1.	To what extent did the medical staff look out for your health?	9.54 (0.89)	9.07 (2.02)	.0590	9.25 (1.57)	8.81 (1.56)	**.0444**
5.	To what extent did you feel that the medical credentials of the medical staff were good for any situation?	9.43 (1.48)	8.88 (2.07)	**.0412**	9.22 (1.40)	8.63 (1.59)	**.0038**
10.	To what extent were you trustful in placing your life in the hands of the hospital's medical staff?	9.45 (1.34)	8.81 (1.68)	**.0050**	9.12 (1.37)	8.56 (1.60)	**.0074**
11.	To what extent is it important for you to choose your physician–caregiver and to know that only she or he is caring for you?	9.53 (1.82)	7.78 (3.23)	**<.0001**	9.82 (0.54)	7.33 (3.50)	**<.0001**
12.	To what extent did the medical staff listen to you and address your questions and concerns?	9.55 (0.94)	9.05 (2.09)	.0525	9.09 (1.74)	8.94 (1.55)	.5028
13.	To what extent did you find the explanations that you received from the medical staff during your hospital stay clear and understandable?	9.54 (0.97)	9.07 (2.16)	.0788	9.21 (1.46)	9.00 (1.50)	.3108
17.	Should it become necessary, to what extent would you recommend this hospital to friends and relatives?	9.32 (1.99)	9.13 (2.06)	.5109	9.31 (1.55)	8.70 (2.26)	**.0186**
*Cluster 2: The patients' concerns and additional considerations during their hospital stay*							
2.	To what extent did you consider your financial situation when you were hospitalized?	2.92 (3.20)	2.14 (2.61)	.0562	2.47 (2.76)	2.19 (2.67)	.4607
3.	To what extent were you concerned that the staff might make a mistake in its medical decisions?	2.07 (2.30)	2.79 (2.75)	.0538	3.19 (3.13)	3.22 (2.92)	.9467
4.	If the staff asks you to take part in a study, to what extent would you fear that they would care more about the study and less about what matters to you?	3.22 (3.30)	3.46 (2.54)	.5591	4.40 (3.54)	3.23 (2.16)	**.0081**
*Cluster 3: The extent of explanation and cooperation between the patient/patient's family and the medical staff*							
6.	To what extent did you feel that the medical staff allowed you to say things that were important to you?	9.08 (1.92)	9.13 (1.95)	.8704	8.93 (1.93)	8.68 (1.84)	.3433
7.	To what extent does the hospital staff make sure that the prescription for medication that you've been given is correct and accurate?	8.81 (2.60)	9.05 (2.19)	.4716	8.31 (3.22)	8.99 (1.86)	.0975
8.	To what extent did the medical staff at the hospital present you with options of care so that you could choose freely?	8.40 (3.02)	8.52 (2.25)	.7415	7.97 (3.25)	8.10 (2.21)	.7398
15.	To what extent did you feel that you knew what the next stage of care would be?	9.25 (1.68)	9.01 (2.18)	.4053	8.70 (2.20)	8.52 (2.22)	.5500
16.	To what extent did you feel that you were co‐opted into decisions and that your preferences were taken into account?	8.73 (2.38)	8.12 (2.39)	.0752	8.24 (2.63)	7.59 (2.54)	.0788
21.	To what extent do you agree with this sentence: ‘The doctor–patient relationship is like a contract/a partnership meant to attain health’?	8.89 (2.33)	9.31 (1.77)	.1362	8.91 (2.15)	9.23 (1.83)	.2770
22.	To what extent do you believe that the patient's family shares responsibility for care?	7.56 (3.56)	8.91 (2.17)	**.0006**	8.08 (3.01)	8.92 (1.94)	**.0281**
23.	To what extent do you agree with this sentence: ‘Four players share responsibility for healthcare: the doctor, the patient, the family, and the hospital’?	9.23 (2.02)	9.07 (2.20)	.5919	8.79 (2.34)	8.97 (2.39)	.5872
*Cluster 4: The way the staff handles medical information*							
9.	To what extent is it important for you that the staff keep your information confidential?	8.96 (2.37)	9.34 (1.67)	.1632	9.18 (2.11)	9.17 (2.02)	.9579
14.	To what extent did you feel that the medical staff coordinated and cooperated with one another in its work (e.g., in sharing information with one another)?	9.66 (0.89)	9.14 (1.94)	**.0300**	8.95 (2.09)	8.86 (1.53)	.7367
*Cluster 5: The commitment strength of medical staff to the patient*							
18.	‘To what extent does the medical staff at the hospital tend to be committed to the patient who makes an effort to be involved in care?’	6.11 (4.12)	8.71 (2.43)	**<.0001**	6.36 (3.89)	8.61 (1.81)	**<.0001**
19.	To what extent does the medical staff at the hospital tend to be more committed to a patient who has a family?	5.14 (4.12)	8.47 (2.65)	**<.0001**	4.84 (4.00)	7.97 (2.69)	**<.0001**
20.	‘To what extent does the medical staff at the hospital tend to be more committed to a well‐educated patient?’	3.53 (3.96)	7.19 (3.33)	**.0000**	3.92 (3.83)	7.29 (3.03)	**.0000**

*Note*: Bold formatting of *p* values indicates significance (*p* < .05).

Abbreviation: SD, standard deviation.

Participants treated at both institutes expressed higher trust scores in placing their life in the hands of the medical staff, with those treated at the private hospital expressing higher mean trust than those treated at the public hospital (9.27 vs. 8.81, *p* = .0007). This difference was also observed among women and among participants 60 years and older, but not among men and younger participants. Education level did not affect this observation.

Participants who had surgery at the private hospital perceived greater importance to choosing their treating physician and to the knowledge that only this physician would be treating them (9.70 vs. 7.75, *p* < .001). This perceived difference between the private and public hospitals was maintained regardless of gender, age group and education level.

Participants in both institutions perceived that the medical staff listened to them and addressed their questions and concerns to a high extent. Participants under 40 years who had surgery at the private hospital gave a significantly higher mean score to this statement compared to their counterparts who had surgery at the public hospital (9.47 vs. 8.57, *p* = .0182), while no differences were seen among older participants.

The participants also reported that the explanations they received from the medical staff during their hospital stay were clear and understandable to a high extent. Women who had surgery at the private hospital gave a higher mean score to this statement compared to women who had surgery at the public hospital (9.47 vs. 8.86, *p* = .0078). No difference was observed for men, by age group or by education level.

### 4.2 Patient concerns and additional considerations during their hospital stay

*The second cluster* focuses on patients' concerns and additional considerations during their hospital stay and includes three questions: concern for medical errors, concern for treatment bias due to research and financial considerations.

Overall, the participants only gave little consideration to their financial situation when they were hospitalized, and no statistically significant difference was observed between participants who had surgery at the private hospital compared to those who had surgery at the public one. Gender, age or education level did not have an effect on this statement.

Participants were only a little concerned that the staff might make a mistake in its medical decisions. Gender, age or education level did not have an effect on this statement.

Overall, participants were only slightly concerned that if the staff asked them to take part in a study, the staff would care more about the study and less about the patient. Here, participants treated at the private hospital were significantly more concerned than those treated at the public hospital (3.98 vs. 3.42, *p* = .0422). In addition, participants 60 years and older and participants with academic education who had surgery at the private hospital were significantly more concerned than those treated at the public hospital (4.14 vs. 2.95, *p* = .0041 and 4.40 vs. 3.32, *p* = .0081, respectively). No difference was observed among younger participants or among those with ≤12 years of education.

### 4.3 The extent of explanation and cooperation between the patient/patient's family and the medical staff

*The third cluster* focuses on the extent of explanation and cooperation between the patient/patient's family and the medical staff. It comprises eight questions: staff respect for patient's preferences and cultural background; supplying information to enable free choice; informing future steps in the patient's journey; shared decision‐making; feeling that patient–physician relationship is like a contract or a partnership meant to attain health; family involvement in sharing responsibility for patient care; the perception of shared responsibility of four healthcare players: the physician, patient, family and hospital and the intent for medication prescription accuracy.

The participants perceived that the medical staff allowed the patients to speak about issues that they (the patients) consider important to a great extent. Hospital type, gender, age or education level did not have an effect on this statement.

The participants perceived to a great extent that the hospital staff made sure that medication prescriptions given to the patients were correct and accurate. Interestingly, participants <40 years who had surgery at a private hospital agreed with this statement to a significantly higher extent than their counterparts who had surgery at the public hospital (9.53 vs. 8.58, *p* = .0377). On the other hand, participants aged 40–59 who had surgery at a private hospital agreed with this statement to a lesser extent than participants of the same age who had surgery at a public hospital (7.36 vs. 9.23, *p* = .0001). No difference in agreement with this statement was observed for participants 60 and older or by education level.

Participants perceived that they could freely choose their care option to a larger extent. Participants younger than 40 who had surgery at the private hospital gave a higher mean score to this statement than participants of the same age group who had surgery at the public hospital (9.02 vs. 8.07, *p* = .0428). No difference was observed for the other age groups, or by gender or education level.

At both hospital types, participants perceived to a high extent that they knew what the next stage of care would be, that they were included in decision‐making and that their preferences were considered. No differences were observed by gender and education level. Only participants younger than 40 who had surgery at the private hospital perceived greater inclusion in decision‐making and preference consideration compared to their counterparts who had surgery at the public hospital (8.86 vs. 7.43, *p* = .0044).

All participants agreed to a high extent that the patient–physician relationship is like a contract or a partnership meant to attain health with no statistically significant difference between hospitals. Gender, age and education level did not affect the extent of agreement with this statement.

The participants also agreed with the sentence that the patient's family shares responsibility for care. Those who had surgery at a public hospital agreed more with this sentence than those who had surgery at a private hospital (8.61 vs. 7.95, *p* = .0118). Similarly, men who had surgery at a public hospital and participants who were younger than 40 agreed more with this sentence than those who had surgery at the private hospital (8.88 vs. 7.62 vs, *p* = .0013 and 8.97 vs. 7.82, *p* = .0339), while no difference was observed for women, or patients 40 years or older. Participants who had surgery at a public hospital agreed more with this sentence than those who had surgery at a private hospital, regardless of their education level.

The participants also agreed to a large extent that the physician, patient, family and hospital share the responsibility for healthcare. Gender, age or education level had no effect on this statement.

### 4.4 Handling of medical information by staff

*The fourth cluster* comprised two questions that focus on the way the staff handles medical information: confidentiality and information transfer among staff members (handover).

The participants considered it highly important that the personnel keep their medical information confidential and that the medical staff coordinated and cooperated with one another while working. No differences were found between those who had surgery at the private hospital compared to those who had it at the public hospital. Gender, age or education level had no effect on these statements.

## 5 COMMITMENT OF MEDICAL PERSONNEL TO THE PATIENT

*The fifth and last cluster* comprises three questions that illuminate the commitment strength of medical staff to the patient based on individual characteristics, that is, perceived bias to more involved patients, patients with supportive families or well‐educated patients.

Participants who had surgery at the public hospital perceived to a significantly greater extent than those who had surgery at the private hospital that the medical staff at the hospital tend to be more committed to patients who make an effort to be involved in care (8.46 vs. 6.65, *p* < .0001), to patients with families (8.05 vs. 4.97, *p* < .0001) and to well‐educated patients (6.91 vs. 3.73, *p* < .0001). The same significant trend was observed when the analysis was done by gender, age (all age groups) and education level (both education levels).

## 6 DISCUSSION

Patient preferences affect their choice of private or public health providers. These preferences are influenced by individual, sociodemographic and cultural characteristics as well as by the characteristics of the health service provider.^24^ Our analysis revealed four main themes, based on the patient's experience that were associated with patient preferences: (1) physical conditions and the hospital environment including cleanliness, (2) hospital policies concerning personal empowerment and patients' perceived autonomy, (3) whole‐person care, a good patient experience and (4) communication among care teams, and between patients and care teams using words that patients can understand, responsiveness and attentiveness to needs.^25^, ^26^

Israel has a public healthcare system, but there are a few private hospitals that individuals may choose to approach through private insurance coverage or out‐of‐pocket payment.^16^ Our aim was to expose the main incentives for these choices. Among the leading reasons suggested were swift accessibility, improved facilities and service as well as the option to choose a specific expert. Alongside, we assume that barriers to choosing a private hospital may be high expenses and lack of former relationship with the surgeon and the medical staff.

An in‐depth examination of the findings pointed to the important role of sociodemographic parameters in shaping patients' experiences and preferences. Interestingly, differences in several sociodemographic parameters were observed, as patients of the private hospital were older and with higher levels of education than those of the public hospital. It should be noted that this finding might also correlate with the economic resources available for the two populations. In England, less deprived patients are more likely to be treated at an independent sector provider and suggested that this may be partly due to the provider location and partly due to cultural and social barriers that prevent poorer patients to consider for‐profit providers.^27^ Additionally, patients who underwent elective surgery in independent sector treatment centres were healthier, had less severe preoperative symptoms and were more affluent compared to those who underwent the same procedures in National Health Services hospitals.^28^, ^29^ Similarly, reforms that encouraged the entry of private primary care provision in Sweden mostly benefitted patients with above‐median incomes and those who lived in urban areas.^30^

The emerging role of age, gender and education in determining health status and patients' expectations were reported in several studies.^31^, ^32^, ^33^ Our analysis revealed similar experiences for both men and women while exploring their perceptions on medical staff's credentials with significantly higher scores given by those treated at the private hospital. A possible explanation might be the perception that the private sector is obliged to rigorous quality control and measures of performance effectiveness. Indeed, perceived quality of care, as well as previous good experience, waiting time and gap payment were the most frequent and important recurrent themes that were cited and related to patients' decision‐making process when choosing between public and private emergency departments in Australia.^34^, ^35^

Notably, women who were treated at the private hospital scored higher to the extent of explanation and cooperation among patients, their families and the medical staff. This trend was not observed in men who had surgery at a private hospital. Our results corroborate the findings of Chandra et al.,^36^ who demonstrated that gender was one of the main factors that was significantly associated with trust. They also pointed to the importance of medical teams' interpersonal and communication skills in relation to increasing patients' trust and improved health outcomes. Another cluster that revealed a significant difference between the perceptions of men and women dealt with medical staff's commitment to patients, with significantly higher scores given by patients that had surgery at the public hospital (Table 2). This finding might be related to inherent differences in the interactions between the patient and the medical teams in the two healthcare systems.^37^ For example, the presence of interns in the public health system may help in the communication between patients and medical staff in contrast to the private healthcare system, which does not train interns. This finding is also in line with another study that pointed to patients' need for staff's commitment to establish trust in the medical team.^38^

Our finding that patients expressed greater trust in the public healthcare system contrasts with the findings of a previous study conducted in Israel, which showed an association between prolonged waiting times in the public healthcare system and lower trust levels.^19^ We postulate that the longer interaction with medical teams working in the public health system, their interest and expanded dialogue with patients all play a major role in the patient's journey, and are reflected by higher trust levels in medical staff's commitment. In a study conducted in South Australia, longer waiting times for public hospital services did not lead to widespread distrust in public hospitals or healthcare professionals because the patients blamed this on an underfunded system and over‐worked staff, while doctors and nurses were perceived as doing their best and therefore were considered trustworthy. In contrast, private hospital patients generally distrusted public hospitals. The authors suggested that in public hospitals institutional trust is based on basic expectations of consistency and minimum standards of care and safety, while trust in the private hospital may be based on the additional and higher‐level expectations of flexibility, reduced waiting and more time with healthcare professionals.^39^

Stratification of the responses to the questionnaire by age revealed that young patients scored higher in the extent of explanation and cooperation between the patient/patient's family and the medical staff. This difference may be partly explained by higher orientation and increased health literacy among younger population^40^, ^41^ in comparison to older patients. In a survey conducted in Sweden, younger individuals and those with higher education demanded a more active part in the process of medical decision‐making.^42^ Furthermore, continuously giving relevant information before and during treatment was found to have a positive influence on the choice of healthcare provider.^24^

In a systematic review that examined factors influencing patient choice of surgical care, hospital reputation was the most important hospital‐related factor for patients choosing a location for surgery. Patients wanted hospitals that were viewed as providers of superior care or that were valued highly by the community. In addition hospital atmosphere, which includes interactions between staff and patients, was also considered an important factor in selecting a location for surgical care, even more than information on surgical success and adverse outcomes.^43^ Our findings showed that patients who underwent surgery at the private hospital scored higher items that included freedom of choice and trust in the medical staff while patients who had surgery at the public hospital scored higher items examining commitment to patients and their families and to those dealing with the dialogue and the interaction between patients and medical teams. Therefore, while physical conditions may be better in private hospitals in developed health systems, hospital patient autonomy and patient‐staff communication policies may be emphasized in both public and private systems. Thus, perceived patient‐centeredness may be driven by hospital policies regardless of ownership and funding. Nevertheless, in our study, perceived staff commitment was higher in the private sector, which was probably driven by hospital strategy. Hospital high ratings based on items on the Hospital Consumer Assessment of Healthcare Providers actually emphasize quality of care, however, patients' perceptions and choice of provider may be influenced by commercial data and hospital rating, regardless of accurate outcomes.^44^

## 7 STRENGTHS AND LIMITATIONS

To the best of our knowledge, this is the first study that examines dimensions of patient's trust between the public and private health systems in Israel, highlighting the importance of patient's autonomy, patient preferences and the need for staff commitment to establish a better patient–physician relationship regardless of hospital ownership and funding. The study has several limitations: first, it reflects the findings of only two hospitals: one public and one private. Second, the questionnaire used in this study relates to how care was experienced, rather than what was anticipated; the choice of the health provider (public or private) was made before the service was experienced, thus posing a limitation on data analysis and interpretation. Nevertheless, the retrospective methodology used in our study can assist in identifying factors influencing decision‐making about where to get care within the local health system Third, due to technical reasons, only 10 procedures were examined during the study period. The selected procedures were from various medical fields (otorhinolaryngology, orthopaedics, general surgery, etc.) and therefore they are a reliable presentation of the current situation. However, to reflect the actual snapshot of patient trust in the healthcare system, a further investigation should include most procedures within the healthcare system and should be expanded to additional public and private hospitals in Israel and abroad. In addition, we will initiate a study that will try to identify factors influencing patient's meaning of choices before the decision regarding the health system setting (e.g., private vs. public)

## 8 CONCLUSIONS

This study serves as a pioneer study that points to acute strengths and weaknesses of the private and the public healthcare systems, with relation to patient trust. Patient's preferences are affected by their gender, age and education level as well as by the hospital's physical conditions, their personal empowerment and perceived autonomy, their experience and by patient–provider communication and encounters. A broader study that would include additional hospitals and medical procedures is warranted.

## AUTHOR CONTRIBUTIONS

Royi Barnea made substantial contributions to the conception and design, acquisition of data and has been involved in writing the manuscript and revising it critically for important intellectual content. Aviad Tur‐Sinai made substantial contributions to the conception and design, acquisition of data and has been involved in writing the manuscript and revising it critically for important intellectual content. Osnat Levtzion‐Korach has been involved in drafting the manuscript and acquisition of data and revising it critically for important intellectual content. Yossi Weiss has been involved in drafting the manuscript and acquisition of data and revising it critically for important intellectual content. Orna Tal made substantial contributions to the conception and design, acquisition of data and has been involved in writing the manuscript and revising it critically for important intellectual content. All authors read and approved the final manuscript.

## CONFLICTS OF INTEREST

The authors declare no conflicts of interest.

## Data Availability

The datasets used and/or analyzed during the current study are available from the corresponding author on a reasonable request.
